# LONG-TERM CARE TRAJECTORIES AFTER STROKE IN SWEDEN AND THE UNITED STATES

**DOI:** 10.1093/geroni/igae098.3587

**Published:** 2024-12-31

**Authors:** Karin Modig, Greg Arling

**Affiliations:** Karolinska Institutet, Stockholm, Stockholms Lan, Sweden; Purdue University, West Lafayette, Indiana, United States

## Abstract

Stroke is a major cause of mortality and disability in old age worldwide, often requiring ongoing formal long-term care (LTC). Only a handful of studies examining longitudinal patterns of institutional and community based LTC beyond the immediate post-stroke period. In order to better understand the impact of LTC use by stroke survivors, we compared findings from studies of LTC trajectories among people discharged from the hospital after a stroke in Sweden and the United States. The Swedish study modeled LTC trajectories over a three-year period for a nationally representative cohort of 31,560 stroke survivors age 70 and older during 2015 to 2017, while the US study modeled LTC trajectories over 12 months among a sample of 3,811 Veterans discharged from Veterans Administration (VA) hospitals after stroke during 2007. Approximately half of the sample in each study was alive and without a history of LTC use at one year. Use of LTC in both studies was associated with LTC use prior to stroke, advanced age, number of comorbid conditions including dementia, and frailty/dependency. In the Swedish study, transitions into formal LTC tended to be unidirectional - few people entering formal LTC ever returned to no care, and few people entering care homes ever transitioned back to home care. In the VA study, frequent transitions occurred between nursing homes and home-based LTC, as well as out of the LTC system entirely. We discuss how differences in LTC policies, particularly the role of the nursing home, affect LTC trajectories in the two countries.

